# Is there an association between diabetes and neck and back pain? A systematic review with meta-analyses

**DOI:** 10.1371/journal.pone.0212030

**Published:** 2019-02-21

**Authors:** Daniel Pozzobon, Paulo H. Ferreira, Amabile B. Dario, Lisandra Almeida, Giovana Vesentini, Alison R. Harmer, Manuela L. Ferreira

**Affiliations:** 1 Institute of Bone and Joint Research, The Kolling Institute, Sydney Medical School, University of Sydney, Sydney, NSW, Australia; 2 Musculoskeletal Health Research Group, Faculty of Health Sciences, University of Sydney, Sydney, NSW, Australia; 3 School of Public Health, Sydney Medical School, University of Sydney, Sydney, NSW, Australia; 4 Discipline of Physiotherapy, Institute of Health Sciences, Federal University of Bahia, Salvador, Brazil; 5 Department of Gynaecology and Obstetrics, Botucatu Medical School, UNESP–Paulista State University, Botucatu, São Paulo, Brazil; Augusta University, UNITED STATES

## Abstract

**Background and objective:**

Approximately half of the population will experience either low back pain or neck pain, at some point in their lives. Previous studies suggest that people with diabetes are more likely to present with chronic somatic pain, including shoulder, knee and spinal pain. This study aimed to systematically review and appraise the literature to explore the magnitude as well as the nature of the association between diabetes and back, neck, or spinal (back and neck) pain.

**Databases and data treatment:**

A systematic search was performed using the Medline, CINAHL, EMBASE, and Web of Science electronic databases. Studies which assessed the association between diabetes and back or neck pain outcomes, in participants older than 18 years of age were included. Two independent reviewers extracted data on the incidence of pain and reported associations.

**Results:**

Eight studies were included in the meta-analyses. Meta-analyses showed that people with diabetes are more likely to report low back pain [5 studies; n: 131,431; odds ratio (OR): 1.35; 95% Confidence Interval (CI): 1.20 to 1.52; p<0.001] and neck pain (2 studies; n: 6,560; OR: 1.24; 95% CI: 1.05 to 1.47; p = 0.01) compared to those without diabetes. Results from one longitudinal cohort study suggested that diabetes is not associated with the risk of developing future neck, low back or spinal pain.

**Conclusions:**

Diabetes is associated with low back and neck individually, and spinal pain. The longitudinal analysis showed no association between the conditions. Our results suggest that diabetes co-exists with back pain; however, a direct causal link between diabetes and back pain was not established.

**Systematic review registration:**

PROSPERO registration CRD42016050738.

## Introduction

Low back and neck pain are commonly reported musculoskeletal disorders. Approximately 80% of adults will experience low back pain, and 47% will experience neck pain, at some point in their lives [1]. Similarly, diabetes mellitus is an increasingly prevalent chronic condition, with an estimated 382 million people living with this metabolic disease around the world [2]. Previous data suggest people with diabetes are more likely to present with chronic somatic pain, including painful diabetic peripheral neuropathy [3]. For example, a recent cohort study with more than 39,000 participants found that people with diabetes have a significantly higher risk of developing musculoskeletal pain, including back and neck pain [4]. The burden of having both diabetes and musculoskeletal pain, which results in higher levels of pain, disability and psychological distress, is substantially more compared to having just one of the conditions [3, 5].

However, the link between musculoskeletal pain and diabetes remains unclear. Past research suggests that having diabetes may predispose patients to develop lumbar disc disease as a result of secondary, diabetes-related microangiopathy of the lumbar disks [6]. Additionally, the accumulation of advanced glycation end-products (AGE) in animal models of diabetes is linked to increased rates of catabolic reactions in intervertebral disc cells, reduced disc hydration and increased disc tissue stiffness, which leads to intervertebral disc degeneration [7]. However, these findings have not been confirmed in human studies.

Diabetes and back pain also share common risk factors such as obesity [8], physical inactivity [9, 10]; or predisposing factors such as low-grade systemic inflammation [11, 12]. People with a higher body mass index (>35) at a younger age (i.e. 18 years old) are over nine times more likely to develop diabetes later in life [13]. Likewise, obesity is an independent predictor of severe low back pain in the general population [14]. People with diabetes are also less likely to participate in regular physical activity and therefore at a higher risk of developing chronic musculoskeletal conditions, such as back and neck pain [15].

Although, in theory, there are strong links between the two conditions, the nature and magnitude of the association between spinal pain and diabetes is still unclear. The aim of this review, therefore, is to identify and appraise the literature on the association between diabetes and back, neck or spinal pain. The findings will have a substantial impact on the management and prevention of these two highly burdensome conditions.

## Methods

### Data sources and searches

This systematic review was conducted in accordance with the PRISMA guidelines [16] and was prospectively registered on PROSPERO (protocol number CRD42016050738) (S1 Text). A systematic electronic search was performed using the following databases, including automated updates, from inception to October 2017: MEDLINE, EMBASE, CINAHL, and Web of Science. To identify potential studies, we used keywords related to the exposure (e.g. diabetes, insulin resistance), outcome (e.g. back pain, neck pain) and study design (e.g. cohort study, incidence) (S1 Table). The first screening of potentially relevant records was conducted by one author (DP) based on titles and abstract, and two authors (DP and LA) independently performed the final selection of included trials based on full-text evaluation. A third reviewer arbitrated in cases of disagreement (MLF). The reference lists of included papers were also checked to ensure that all eligible studies were identified.

### Study selection

Cross-sectional, case-control, twin-control and cohort observational studies that evaluated the associations between type 1 or type 2 diabetes and non-specific back, neck or spinal pain were included. To be eligible, studies had to be full reports and include participants older than 18 years of age with the conditions of interest (diabetes and non-specific back pain, neck pain or both).

Studies were excluded from this review if they: i) included sites of pain other than back or neck and did not present separate results for the symptom of interest; ii) included patients with severe spine pathology (e.g., cancer, fracture, infectious bone disease); iii) included data on multiple diseases and did not present separate results for association between diabetes, back or neck pain; or iv) were not written in English. Randomised controlled trials, single-case studies and animal studies were also excluded, as were those focusing on gestational diabetes, pre-diabetes and any other type of non-type 1 or 2 diabetes.

### Data extraction

A standardized data extraction form was used by two independent reviewers (DP and LA) to gather data from the included studies on study design, sample characteristics, back and neck pain, diabetes and associations of interest. A third author (MLF) resolved disagreements in data extraction. Authors from published studies were contacted to request additional information or data that were not reported in the original articles.

### Methodological quality assessment

The methodological quality of included studies was assessed by two independent reviewers (DP and GV) using the Newcastle-Ottawa Scale (NOS)[17] as recommended by the Cochrane Collaboration [18]. The NOS for assessing cohort studies consists of eight items grouped into three categories, namely: selection, comparability, and outcome. A star system, ranging from zero to eight stars, is used to classify the quality of the study being reviewed (the more stars the study receives in each category, the higher its methodological quality). Based on previous studies [19], an adapted version of the original scale was used to assess cross-sectional studies. After the independent assessment of included studies by reviewers, each study received the following categorical score representing its quality: good (3 or 4 stars in selection domain AND 1 or 2 stars in comparability domain AND 2 or 3 stars in outcome domain), fair (2 stars in selection domain AND 1 or 2 stars in comparability domain AND 2 or 3 stars in outcome domain) or poor (0 or 1 star in selection domain OR 0 stars in comparability domain OR 0 or 1 star in outcome domain).

### Data analysis

Meta-analyses were performed to assess the associations between predictor groups (i.e. participants with diabetes and participants without diabetes) and outcomes of interest (i.e. back or neck pain), using a random effects model. Similar studies were grouped according to site of pain [i.e. low back, neck or spinal pain (concurrent low back and neck pain)] and study design (cross-sectional or longitudinal). The pooled associations are expressed as the odds ratios (with 95% confidence intervals) for back, neck, or spinal pain separately, and presented by study design (i.e. cross-sectional or longitudinal). Between-study heterogeneity was calculated using I^2^ (I^2^ <25%: small heterogeneity; 25% <I^2^< 75%: moderate heterogeneity; I^2^> 75%: large heterogeneity). All meta-analyses were conducted using Comprehensive Meta-Analysis software (Comprehensive Meta-Analysis, Englewood, New Jersey, US).

Funnel plots were built for each meta-analysis to assess possible publication bias. We plotted the precision (i.e. standard error) of included studies against the estimates of association and results were visually inspected. Egger’s test was used to quantify plot asymmetry, for which a null hypothesis represents symmetry of the plotted data.

## Results

**Fig 1:**
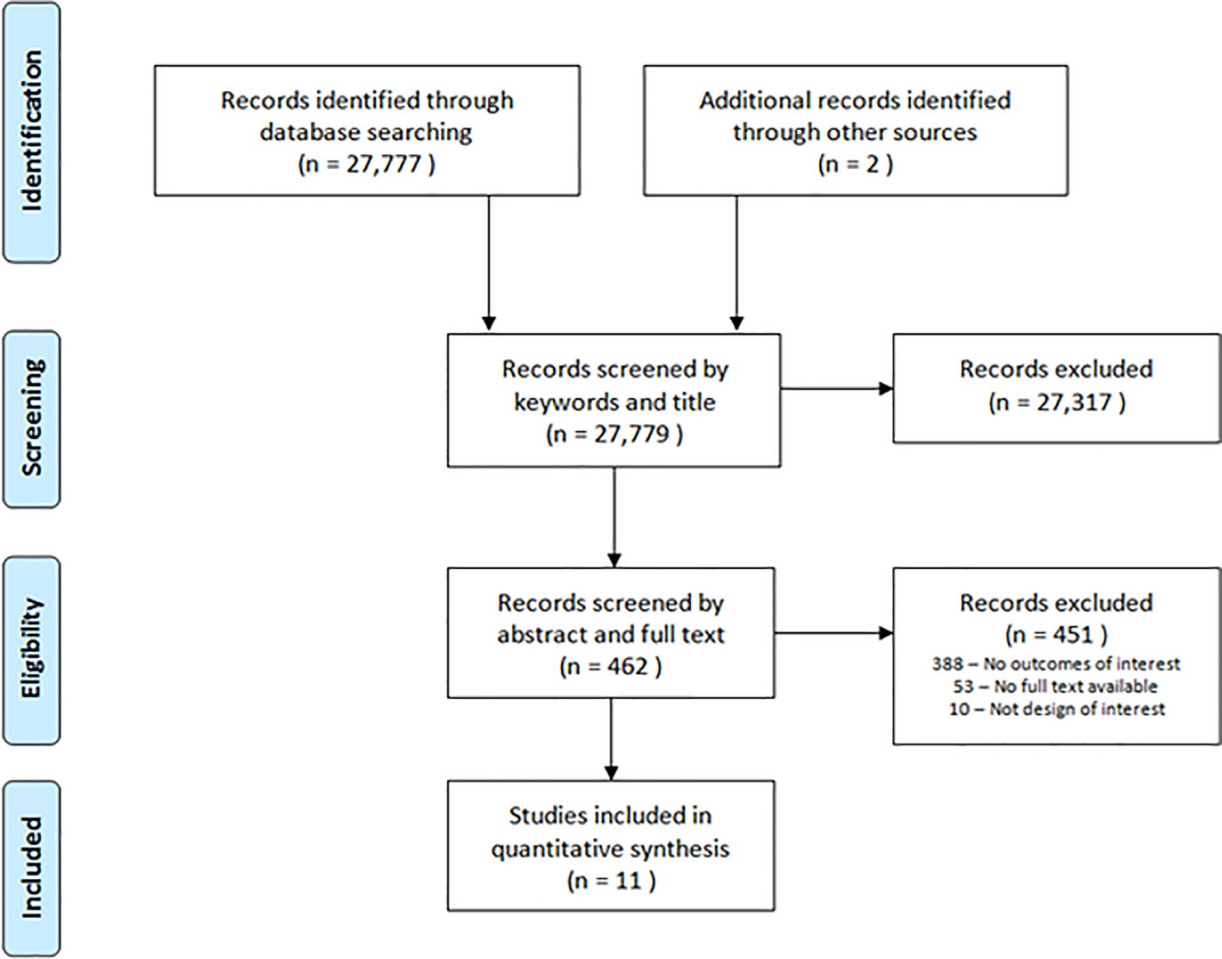
Screening flowchart. Flowchart of search strategy and screening process.

The search identified a total of 27,779 studies. After removing duplicates and screening titles, 462 abstracts were assessed for inclusion. A total of 451 abstracts were excluded, leaving 11 studies to be included in the review [20–30] (Fig 1).

### Included studies

Eleven studies [20–30] that included 165,445 participants assessed the association between diabetes and neck, back or spinal pain. Studies reported data from six different countries: Canada [29], Finland [26], Iran [21], Spain [20], Denmark [22, 23, 27] and the United States [24, 25, 28, 30]. Sample characteristics, study design and estimates for the associations of each study are presented in Table 1. The authors defined neck and back pain as having pain, aching or stiffness in the neck or back on most days. Participants were considered to have diabetes if they reported the condition during the first interview either with or without a health professional diagnosis. Two studies included only participants with type 2 diabetes [20, 27], however, the remainder of the included studies did not report the type of diabetes, and none of the included studies presented disaggregated results for type 1 and type 2 diabetes. All studies presented results from cross-sectional analyses investigating the association between diabetes and a range of outcomes related to back or neck pain, including lifetime prevalence [20–30], pain severity [20, 27] and hospital admissions related to back pain [28]. One study also presented longitudinal data on the association between diabetes and neck, back and spinal pain [20].

**Table 1:** Included studies.

Author, year	Study sample	Design	Assessment of Diabetes	Assessment of Spinal Pain	Results	Quality Score
Dario, 2017	N = 2,096	Cross-sectional and longitudinal twin design	Self reported	Care seeking for chronic lower back or neck pain	After adjusting for age, sex, work-related physical activity, BMI and smoking, diabetes **was associated with neck pain (OR 1.37; 95% CI 1.01 to 1.85, n = 2,074) and severe neck pain (OR 2.28; 95% CI 1.24 to 4.21, n = 1,511) in cross-sectional analyses.** No association was observed in longitudinal analyses of mild (OR 1.16; 95% CI 0.65 to 1.91, n = 1,111) or severe neck pain (OR 1.91; 95% CI 0.52 to 6.95, n = 138).	Good
	Mean Age = 53.6 ± 7.3 years, with type 2 diabetes.				Diabetes was not associated with mild or severe low back pain in cross-sectional (OR 1.18; 95% CI 0.86 to 1.60; n = 2,084 and OR 1.63; 95% CI 1.0 to 2.64; n = 1,525, respectively) or longitudinal analyses (OR 0.84; 95% CI 0.51 to 1.40; n = 1,077 for mild and OR 1.91; 95% CI 0.67 to 5.46; n = 218 for severe low back pain).	
	Duration of diabetes: not reported.				Diabetes did not increase the risk of developing spinal pain or severe spinal pain after 2 to 4 years (n = 1,284, OR 0.85, 95% CI 0.42 to 1.73 and n = 98; OR 3.67, 95% CI 0.84 to 16.03, respectively).	
Eivazi, 2012	N = 417	Cross-sectional	Self reported	Self-reported low back pain in the last 12 months	**The group with diabetes had a higher incidence of low back pain than the group without diabetes (63.4% and 47%, respectively, p = 0.009).**	Poor
	Mean Age = 52.5 years old, with diabetes.					
	Duration of diabetes: not reported.					
Hartvigsen, 2003	N = 4,484	Cross-sectional twin design	Self reported	Self-reported back pain in the last month	After adjusting for sex and age, diabetes was not associated with back pain (OR 1.15; 95% CI 0.94–1.42).	Fair
	Mean age = 81 years old.					
	Duration of diabetes: not reported.					
Hartvigsen, 2004	N = 4,486	Cross-sectional twin design	Self reported	Prevalence of back or neck pain in the last month	After adjusting for age, sex and non-independence of twins, diabetes was not associated with back pain (OR 1.15; 95% CI 0.94–1.42) or neck pain (OR 1.19; 95% CI 0.98–1.47).	Fair
	Age range: 70 to 102 years old.					
	Duration of diabetes not reported.					
Hassoon, 2017	N = 5,106	Cross-sectional	Self reported	Chronic low back pain	After adjusting for age, sex, race, education, income, smoking, physical activity and BMI, **diabetes was associated with chronic low back pain (OR 1.38; 95% CI 1.02–1.92, p = 0.041).**	Good
	Age = 43 ± 13.7 years old, with diabetes.					
	Duration of diabetes not reported.					
Hinyard, 2016	N = 3,645	Cross-sectional	Self reported	Chronic low back pain	After adjusting for age, sex, race and comorbidities, **a higher proportion of the group with diabetes had chronic low back pain than the group without diabetes (25.3% and 16.5%, p<0.0001, respectively).**	Good
	Age = 59.8 ± 14 years old, with diabetes.					
	Duration of diabetes not reported.					
Mäkela, 1991	N = 7,217	Cross-sectional	Self reported	Chronic neck syndrome	After adjusting for age and sex, diabetes was not associated with chronic neck pain (OR 1.04; 95% CI 0.78–1.39).	Fair
	Age: ≥ 30 years old, with neck pain for over 3 months.					
	Duration of diabetes not reported.					
Molsted, 2012	N = 3,874	Cross-sectional	Self reported	Self-reported prevalence of neck or low back pain in the last 14 days	Musculoskeletal pain **was consistently more prevalent** in participants with diabetes when compared to the general population.	Poor
	Age = 60 ± 10 years old, with type 2 diabetes					
	Duration of diabetes 4 ± 4.3 years.					
Ritzwoller, 2006	N = 16,567	Cross-sectional	Medically diagnosed	Hospital admission after a low back pain event	After adjusting for age and sex, **diabetes was associated with a higher risk of hospital admission after a low back pain event (OR 2.02; 95% CI 1.69–2.40).**	Good
	Age = 51.1 years old, with low back pain.					
	Duration of diabetes not reported.					
Slater, 2011	115,915	Cross-sectional	Self reported	Low back pain for the last 6 months	After adjusting for age, sex, income, education, race, BMI and multimorbidity, **diabetes was associated with back problems (OR 1.36; 95% CI 1.28–1.45).**	Good
	Age: ≥ 20 years old.					
	Duration of diabetes not reported.					
Wright, 2016	N = 1,638	Cross-sectional	Self reported	Neck pain	After adjusting for age, sex, BMI and race, **diabetes was associated with mild neck pain (OR 1.6; 95% CI 1.1–2.3)** but not moderate/severe neck pain (OR 1.0; 95% CI 0.7–1.5).	Good
	Age = 68 years old, with neck pain.					
	Duration of diabetes not reported.					

Characteristics of included studies. Bold denotes significance at the 0.05 level.

### Methodological quality

Six studies (55%) were rated as having good methodological quality (i.e. score of 6 or more) [20, 22, 23, 25, 28, 29], three studies (27%) were rated as fair (i.e. score 5) [24, 26, 30] and the remaining two studies (18%) [21, 27] were rated as poor (score of 2). All studies had satisfactory sample sizes, nine studies (82%, [20, 22–26, 28–30]) conducted adjustments for potential confounders (e.g. age or sex); eight studies (73%, [20, 22–24, 26, 28–30]) clearly described the statistical analyses utilized and five studies (45%, [20, 22, 23, 25, 28]) conducted retrospective analyses of secure records (S1 Fig).

### Assessment of publication bias

No evidence of small study bias was observed for the studies included in our pooled analyses of low back and neck pain. The evidence was confirmed through visual inspection of funnel plots and the results of Egger’s test (β 2.03; 95% CI -5.16 to 9.23, p = 0.51; S2 Fig). Due to the low number of studies in the severe neck pain and spinal pain meta-analysis (<4), it was not possible to conduct funnel plots to ascertain publication bias.

### Association between diabetes and low back pain

**Fig 2:**
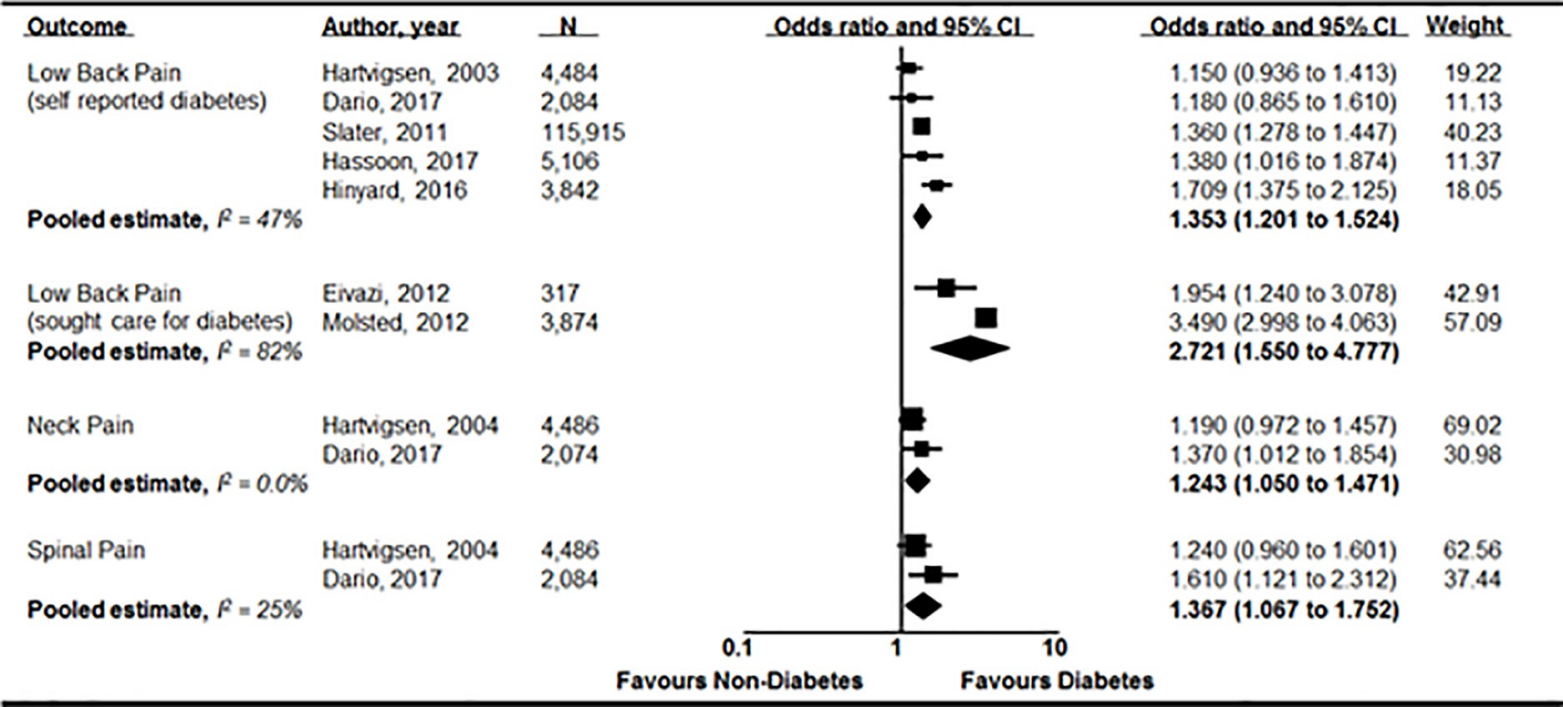
Meta-analysis of included studies. Pooled odds ratios and confidence intervals for the associations between diabetes and back, neck and spinal pain in cross-sectional studies.

Seven cross-sectional studies [20, 22, 24, 25, 27–29] investigated the association between diabetes and low back pain and five of these [20, 22, 24, 25, 29] presented enough data to be pooled. Our pooled analysis showed that diabetes was significantly associated with low back pain (n = 131,431; OR 1.35; 95% CI 1.20 to 1.52, p<0.001; I^2^ = 47%) (Fig 2). Data from two other studies [21, 27] that recruited participants with low back pain who sought health care for diabetes were also pooled, and the association between diabetes and low back pain was found to be statistically significant (n = 4,191; OR 2.72; 95% CI 1.55 to 4.78, p<0.001; I^2^ = 82%)(Fig 2). One study [20] investigated the association between diabetes and severe low back pain and found a significant association with adjusted analysis (n = 1,525; OR 1.63; 95% CI 1.0 to 2.64). Only one longitudinal study [20] investigated whether diabetes at baseline increased the risk of future chronic or severe low back pain after two years; and no significant association was evident (n = 1,077; OR 0.84; 95% CI 0.51 to 1.40 and n = 218; OR 1.91; 95% CI 0.67 to 5.46, respectively).

One study [28] assessed the association between diabetes and back pain and found diabetes increased the likelihood of an inpatient admission in the two years subsequent to the index back pain event by 102% (95% CI 1.69 to 2.40).

### Association between diabetes and neck pain

Four cross-sectional studies [20, 23, 26, 30] investigated the association between diabetes and neck pain, and two of these [20, 23] presented enough data to be pooled. Our pooled analyses showed that diabetes was associated with neck pain (n = 6,560; OR 1.24; 95% CI 1.05 to 1.47; p = 0.01; I^2^ = 0%) (Fig 2). One study [20] investigated the association between diabetes and severe neck pain and found a significant association in both unadjusted (n = 1,515; OR 2.11; 95% CI 1.17 to 3.79) and adjusted (n = 1,515; OR 2.28; 95% CI 1.24 to 4.21) analyses. The same paper [20] reported data from longitudinal analysis and showed that diabetes was not associated with increased risk of developing neck pain (n = 1,111; OR 1.16; 95% CI 0.65 to 1.91) or severe neck pain (n = 138; OR 1.91; 95% CI 0.52 to 6.95) after two years follow-up.

One study [26] assessed the association between chronic neck syndrome and diabetes in adults and the association was not found to be statistically significant (n = 412; OR 1.04; 95% CI 0.78 to 1.39). One study [30] assessed the association between diabetes and mild or moderate/severe neck pain and no association was found (n = 154; OR 1.3; 95% CI 0.9 to 1.9 and n = 187; OR 0.8; 95% CI 0.6 to 1.3, respectively).

### Association between diabetes and spinal pain

Two cross-sectional studies [20, 23] investigated the association between diabetes and spinal pain (low back and neck pain). Pooling of data from these studies showed diabetes was associated with spinal pain (n = 6,570; OR 1.37; 95% CI 1.07 to 1.75; p = 0.01; I^2^ = 25%) (Fig 2). One longitudinal study [20] found no association between diabetes and increased risk of developing spinal pain or severe spinal pain (n = 1,284, OR 0.85, 95% CI 0.42 to 1.73 and n = 98; OR 3.67, 95% CI 0.84 to 16.03, respectively).

## Discussion

### Main findings

This review aimed to appraise and summarize the literature regarding the association between diabetes and low back, neck or spinal (both low back and neck) pain. We found eleven studies that investigated the association between diabetes and a range of outcomes related to back, neck and spinal pain.

Our pooled cross-sectional results suggest that people with diabetes are significantly more likely to report back, neck, and spinal pain than those without diabetes; with odds ratios ranging from 1.24 to 2.72. The association with diabetes and back pain was stronger among people seeking care for diabetes (OR 2.72; 95% CI 1.55 to 4.78) (Fig 2). This change could be due to participants having higher average BMI, more severe diabetes (i.e. higher glycated haemoglobin, indicative of poorer glycaemic control) and/or diabetes for a longer duration. If patients seeking care were those with poorer metabolic control, it might indicate that they were more prone to developing more prevalent and more severe associated complications, including back pain.

Our review only identified one longitudinal study assessing the association between diabetes and the development of future neck, low back or spinal pain. The study did not find any evidence of a temporal effect. Together, the findings of our review suggest that there is a positive association between diabetes and neck, low back and spinal pain. However, the currently limited evidence does not support a causal relationship between diabetes and back pain due to a lack of evidence of any temporal effect. Furthermore, the only included study that reported longitudinal analyses had a reduced sample size for the longitudinal analysis and used a follow-up duration of only two to four years, which may be considered short and insufficient to show an effect of time on the association between diabetes and spinal pain.

If diabetes is not a true risk factor for back pain, another possible factor explaining the cross-sectional association may be the underlying presence of obesity. At the age of 18, men with higher BMI (i.e. BMI>35) are over nine times more likely to develop diabetes when compared to underweight men (i.e. BMI<18.5) [13]. People with higher BMI are also 30% more likely to develop chronic low back pain over a period of 10 years [31]. Obesity is likely to predispose people to develop the two conditions via different pathways, including metabolic [e.g. low-grade systemic inflammation [11, 32] and/or dyslipidaemia [33]] and biomechanical (joint loading) [34, 35] mechanisms. However, four of the eleven included studies [20, 24, 29, 30] adjusted their cross-sectional analyses for BMI, of which two [29, 30] found a significant association between low back pain and diabetes, independent of BMI. While BMI may not accurately reflect obesity status in some participants, the significant association suggests that despite the critical role of obesity, it does not fully explain the relationship between diabetes and back pain, requiring other potential confounders to be accounted for.

Some other possible explanations for a cross-sectional relationship between diabetes, neck, low back and spinal pain are also plausible. For example, the biochemical milieu of diabetes, including hyperglycaemia and dyslipidaemia facilitates tissue damage, mainly due to detrimental effects on blood vessels, and it is plausible that its presence may, therefore, be directly linked to pain [36]. Poorly controlled diabetes can also reduce muscle blood flow [37], increase the likelihood of cartilage inflammation [38] and other tissue damage, such as degeneration of intervertebral discs [39] and consequently spinal canal stenosis [40], which are both common causes of low back and neck pain [21]. Previous research reports that participants with diabetes are more likely to be treated for intervertebral disc herniation of both the cervical [41] and lumbar spine [42]. Diabetes is also known to be associated with loss of muscle mass and strength [43] and is associated with an increased risk of sarcopenia, which is also associated with musculoskeletal pain [44]. Despite these associations and our results having shown a direct link between low back, neck or spinal pain and diabetes, there is insufficient evidence to support the notion that diabetes will increase the risk of future low back, neck or spinal pain.

Finally, it is also possible that a low level of physical activity participation is an underlying factor predisposing people to both conditions. Regular physical activity participation is known to decrease the risk of both low back pain [9] and type 2 diabetes [10], especially when combined with diet [45].

### Strengths and limitations of the study

One of the main strengths of this study is the broad search strategy aimed to maximize the identification of possible references to be screened and included. Our review included studies from six countries representing three global regions, increasing the generalizability of our results. However, a limitation of our study is the inability to conduct separate analyses for type 1 and type 2 diabetes, since none of the included studies reported disaggregated data. There was a broad diversity of outcomes investigated in the included studies, and due to these differences with respect to design, basic characteristics of the populations and outcomes assessed, we chose to pool studies according to the pain site (i.e. back or neck pain). Thus our final site-specific results may underestimate the real association between diabetes and back or neck pain.

Some between-study heterogeneity was observed in the pooled analysis of low back pain studies that included participants who sought care for diabetes. The variability between included studies may possibly be explained by study design (e.g. different length of recruitment periods), definitions of low back pain (i.e. self-reported low back pain with or without leg pain or exclusively low back pain) or type of diabetes (e.g. type 2 only or type not disclosed). Moreover, only one of the included studies adjusted their analyses for age and gender, which may also have contributed for the between-trial heterogeneity.

### Directions for future research

The use of medication to better control diabetes was reported by only one included study [25] with the group who used insulin presenting a higher prevalence of chronic conditions, including low back pain. The use of diabetes medication could influence pain, possibly via its effect on glycaemic control (i.e. glycated haemoglobin and glucose levels), which influences macro- and microvascular complications [46] and via the medication’s analgesic properties [47]. Insulin is also known to affect blood flow [48] which can influence muscle loss [49]. The impact of these medications should be further explored in future studies. Given that we could only identify one longitudinal study assessing the association between diabetes and low back or neck pain, future studies should be conducted to confirm these results in larger samples that are representative of the general population. Future longitudinal studies addressing the association between diabetes and low back or neck pain should include the duration of diabetes and pain symptoms. This will provide important information on the nature of the association between the two conditions.

## Conclusion

This review highlights a positive association between diabetes and low back or spinal pain. However, given the lack of evidence from longitudinal studies, it is unknown whether the association is causal. Future studies should aim to elucidate the mechanisms of the association to provide an opportunity to target preventive and management strategies for people with diabetes.

## Supporting information

S1 Checklist Preferred reporting items for systematic reviews and meta-analyses—PRISMA 2009 checklist. (DOC)

S1 Text PROSPERO review protocol. (PDF)

S1 Table A systematic search was conducted in the MEDLINE, CINAHL, EMBASE and Web of Science electronic databases. Articles titles, keywords and abstracts were searched using the following keywords:
(DOCX)

S1 Fig Methodological quality assessment of included studies. (DOCX)

S2 Fig Funnel plot used to assess the presence of publication bias through visual inspection. (DOCX)
