# Supplementary material for: Is there an association between diabetes and neck and back pain? A systematic review with meta-analyses
Source: PLoS One. 2019 Feb 21;14(2):e0212030. doi: 10.1371/journal.pone.0212030 (PMC6383876; doi:10.1371/journal.pone.0212030)
Supplement: S1 Text — (PDF) [file pone.0212030.s002.pdf]

Association between diabetes and neck and back pain – a systematic review

Daniel Pozzobon, Leandro AC Nogueira, Alison Harmer, Paulo H Ferreira, Manuela L Ferreira

Citation

Daniel Pozzobon, Leandro AC Nogueira, Alison Harmer, Paulo H Ferreira, Manuela L Ferreira. Association between diabetes and neck and back pain – a systematic review. PROSPERO 2016 CRD42016050738 Available from: http://www.crd.york.ac.uk/PROSPERO/display_record.php?ID=CRD42016050738

Review question

Is there an association between diabetes and neck and back pain?

Searches

We searched the following electronic databases: MEDLINE, CINAHL, EMBASE and Web of Science from inception to September 2016. Hand searching was also conducted in reference lists of included articles and identified reviews.

Types of study to be included

Cross-sectional, case-control, twin-control and cohort studies. Surveys that have been conducted in person, by phone, internet, mail or a combination of these.

Condition or domain being studied

Back and neck pain, and diabetes are common disorders which negatively impacts both individual and society. Previous research shows that the two conditions often co-occur and when they do, patients face an especially troublesome situation given the combined symptomatic manifestation, the psychological distress and physical disability that usually accompany them. It is still unclear, however, whether these two conditions are in fact associated.

Participants/population

Studies including patients with the characteristics of interest (type I or type II diabetes and non-specific low back pain, neck pain or both), older than 18 years of age, will be included in the systematic review.

Intervention(s), exposure(s)

Inclusion criteria: Cross-sectional, case-control, twin-control and cohort studies will be included in the review. To be included, studies need to assess the association between type 1 or type 2 diabetes and non-specific low back or neck pain.
Exclusion criteria: Studies will be excluded from this review if they: i) include types of pain other than neck or back and do not present separate results for the symptom of interest; ii) include patients with serious pathology of the spine (e.g., cancer, fracture, infectious bone disease); iii) include multiple diseases and do not present separate results for association between diabetes and back and neck pain; and iv) are not written in English. Randomized controlled trials will also be excluded , as well as reviews, single-case studies and research conducted in animal models or those focusing on gestational diabetes.

Comparator(s)/control

Not applicable.

Context

Primary outcome(s)

Association estimates of the relationship between neck or back pain and diabetes.

Secondary outcome(s)

None.

Data extraction (selection and coding)

A standardized data extraction form will be used by two independent reviewers (DP and LN) to extract data on pain, diabetes and general demographic and anthropometric information from the selected papers and a third author (MF) will resolve any disagreement. If necessary authors will be contacted and asked to provide further information or complement the data reported in the paper.

Risk of bias (quality) assessment

Studies will be assessed according to:
- Sampling: at least one of the following: whole target population, randomly selected sample, or sample stated to represent the population;
- Response rate: reasons for non-response described, non-responders described, comparison of responders and non-responders or comparison of sample and target population given;
- Data collected the same way for all participants and in prospective studies at first and following data collections;
– Outcome measures: questionnaire validated, tested for reproducibility;
– Assessment quality: at least one of the following: interview validated, tested for reproducibility;
– Power calculation shown and inclusion of enough participants to show end points.

Strategy for data synthesis

Estimates of association between back or neck pain and diabetes will be extracted from included studies in any format supplied by the authors. Fisher's z effect sizes and 95% confidence intervals for individual studies will be calculated and pooled using the Comprehensive Meta-Analysis software.

Analysis of subgroups or subsets

Analyses will be separately conducted for neck and back pain, as well as for type 1 or type 2 diabetes.

Contact details for further information

Daniel Pozzobon
dpoz5597@uni.sydney.edu.au

Organisational affiliation of the review

University of Sydney
www.sydney.edu.au

Review team members and their organisational affiliations

Mr Daniel Pozzobon. USYD
Dr Leandro AC Nogueira. USYD
Professor Alison Harmer. USYD
Professor Paulo H Ferreira. USYD
Professor Manuela L Ferreira. USYD

Anticipated or actual start date

08 August 2016

Anticipated completion date

01 May 2017

Funding sources/sponsors

None

Conflicts of interest

None known

Language
English

Country
Australia

Stage of review
Review_Ongoing

Subject index terms status
Subject indexing assigned by CRD

Subject index terms
Back Pain; Diabetes Mellitus; Humans; Neck; Neck Pain

Date of registration in PROSPERO
30 November 2016

Date of publication of this version
30 November 2016

Details of any existing review of the same topic by the same authors

Stage of review at time of this submission

Stage	Started	Completed
Preliminary searches	Yes	Yes
Piloting of the study selection process	Yes	Yes
Formal screening of search results against eligibility criteria	Yes	No
Data extraction	No	No
Risk of bias (quality) assessment	No	No
Data analysis	No	No

Versions
30 November 2016

PROSPERO
This information has been provided by the named contact for this review. CRD has accepted this information in good faith and registered the review in PROSPERO. CRD bears no responsibility or liability for the content of this registration record, any associated files or external websites.
