# Supplementary material for: Is there an association between diabetes and neck and back pain? A systematic review with meta-analyses
Source: PLoS One. 2019 Feb 21;14(2):e0212030. doi: 10.1371/journal.pone.0212030 (PMC6383876; doi:10.1371/journal.pone.0212030)
Supplement: S1 Table — Articles titles, keywords and abstracts were searched using the following keywords: (DOCX) [file pone.0212030.s003.docx]

**Supplementary File 1 Table**

A systematic search was conducted in the MEDLINE, CINAHL, EMBASE and Web of Science electronic databases. Articles titles, keywords and abstracts were searched using the following keywords:

| **Diabetes** | **Neck** | **Back Pain** | **Systematic Review** |
| --- | --- | --- | --- |
| Alloxan Diabetes | atlant* | Back Injuries | cohort studies |
| Streptozocin Diabetes | atlanto-accipital joint | Back Muscles | course |
| Streptozotocin Diabetes | atlato-axial joint | backache | cox |
| Adult-Onset Diabetes Mellitus | axis | coccydynia | cox adj3 regre* |
| Autoimmune Diabetes | brachialgia | coccyx | follow-up studies |
| Brittle Diabetes Mellitus | cervical | dorsalgia | incidence |
| diabet* | cervical atlas | low back pain | life table |
| diabet* insipidus | cervical pain | lower back pain | log rank |
| Diabetes Complications | cervical spine | lumbago | logistic |
| diabetes insipidus | cervical vertebrae | lumbar adj pain | predict* |
| Diabetes Mellitus | cervicalgia | lumbar pain | prognos* |
| Gestational Diabetes | cervicodynia | vertebral pain | survival |
| glucose intoleran* | neck adj3 muscles |  | survival analysis |
| glucose intolerance | neck muscles |  | survival rate |
| Hemochromatosis | neck pain |  | time factors |
| hyperglycaemia | neckache |  | twin-control |
| hyperinsulinemia | occip* |  |  |
| impaired glucose toleran* | odontoid process |  | NOT |
| insulin resistance | odontoid* |  | Randomized Controled Trial |
| insulin* depend* | thoracic adj3 spine |  | Review |
| insulin* resist* | thoracic adj3 spine pain |  | Single case |
| insulin* secret* dysfunc* | thoracic adj3 vertebrae |  | Case study |
| insulin? depend* | thoracic vertebrae |  | Animal |
| Insulin-Dependent Diabetes Mellitus | vertebra |  |  |
| Juvenile-Onset Diabetes Mellitus |  |  |  |
| Ketosis-Resistant Diabetes Mellitus |  |  |  |
| Lipoatrophic Diabetes Mellitus |  |  |  |
| lipodystrophy |  |  |  |
| Maturity-Onset Diabetes Mellitus |  |  |  |
| metabolic* syndrom* |  |  |  |
| Nephrogenic Diabetes Insipidus |  |  |  |
| Neurogenic Diabetes Insipidus |  |  |  |
| non insulin* depend* |  |  |  |
| non insulin? depend* |  |  |  |
| non-insulin* depend* |  |  |  |
| non-insulin? depend* |  |  |  |
| pluri metabolic* syndrom* |  |  |  |
| pluri-metabolic* syndrom* |  |  |  |
| Prediabetic State |  |  |  |
| Slow-Onset Diabetes Mellitus |  |  |  |
| Stable Diabetes Mellitus |  |  |  |
| Type 1 Diabetes Mellitus |  |  |  |
| Type 2 Diabetes Mellitus |  |  |  |
