# Supplementary material for: Is there an association between diabetes and neck and back pain? A systematic review with meta-analyses
Source: PLoS One. 2019 Feb 21;14(2):e0212030. doi: 10.1371/journal.pone.0212030 (PMC6383876; doi:10.1371/journal.pone.0212030)
Supplement: S1 Fig — (DOCX) [file pone.0212030.s004.docx]

**Supplementary File 1 Figure**

Methodological quality assessment of included studies.
